# Supplementary material for: Multi-Morbidity and Risk of Breast Cancer among Women in the UK Biobank Cohort
Source: Cancers (Basel). 2023 Feb 11;15(4):1165. doi: 10.3390/cancers15041165 (PMC9953793; doi:10.3390/cancers15041165)
Supplement: Supplementary file 1 [file cancers-15-01165-s001.zip › cancers-2163680-supplementary.pdf]

Supplementary Materials

Results of MCA

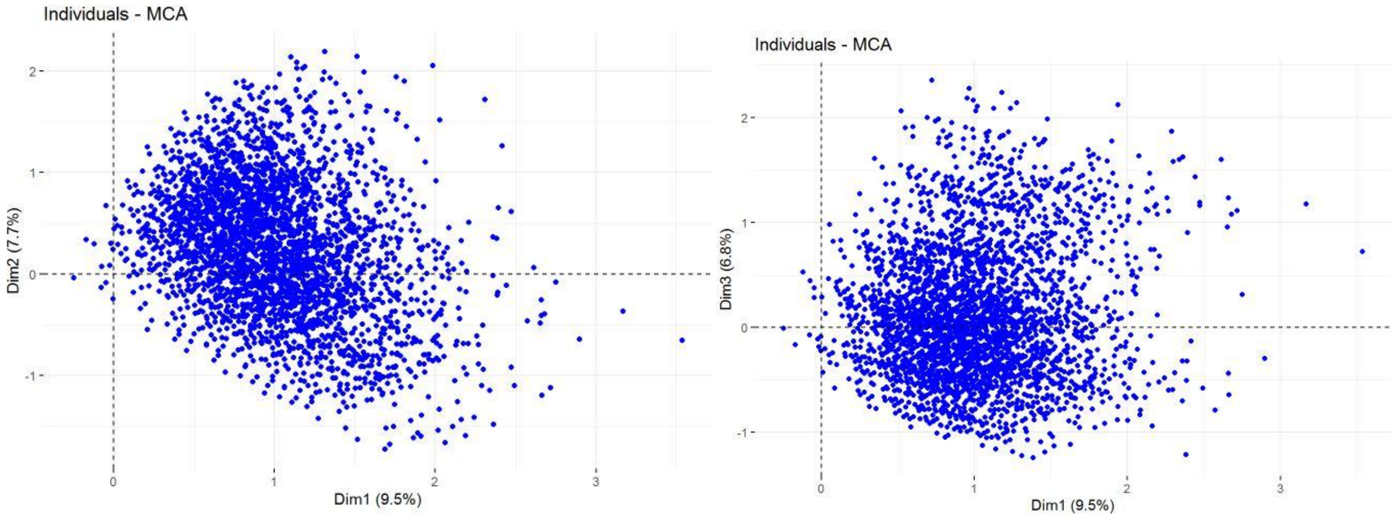

(a) **(b)**

Figure S1: Individuals' point cloud **(a)** on the first two and **(b)** on the first and third axes.

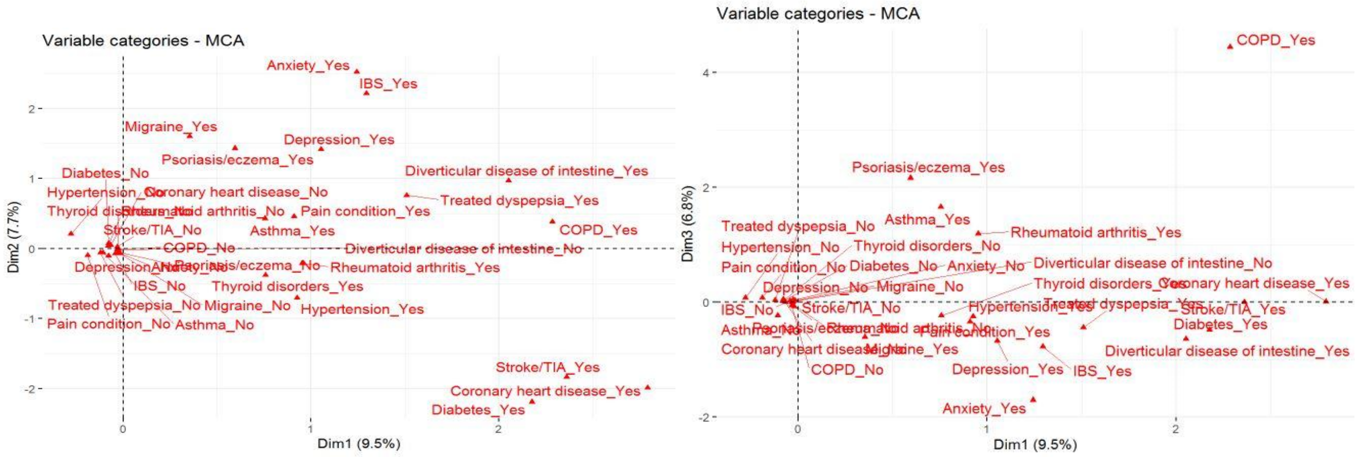

(a) **(b)**

Figure S2: Morbidity categories' point cloud **(a)** on the first two and **(b)** on the first and third axes.

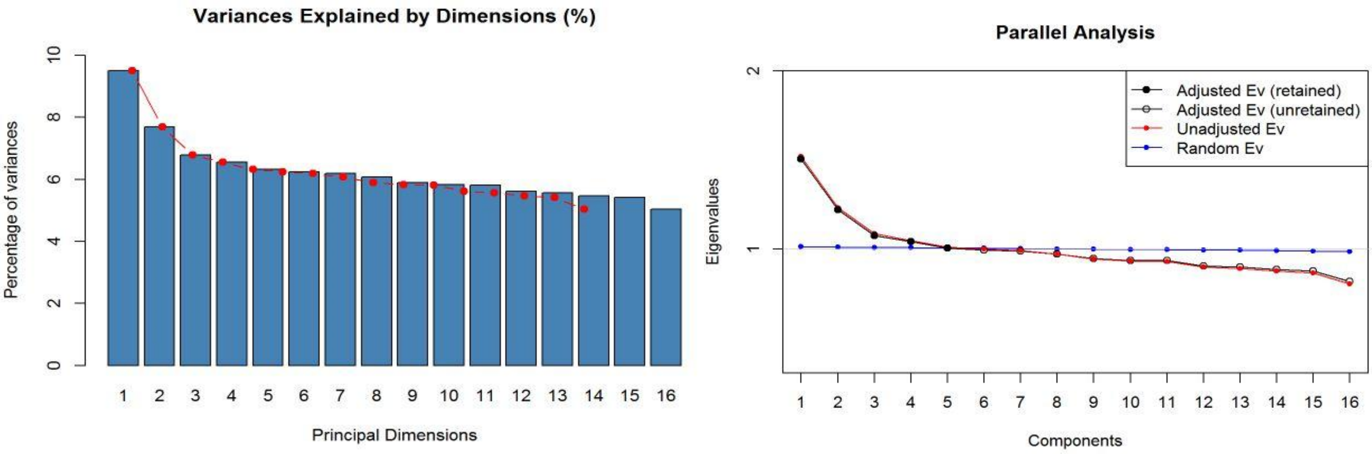

(a) **(b)**

Figure S3: suggested number of MCA dimensions: **(a)** Scree plot; **(b**) Horn's parallel analysis.

Results of pattern analysis

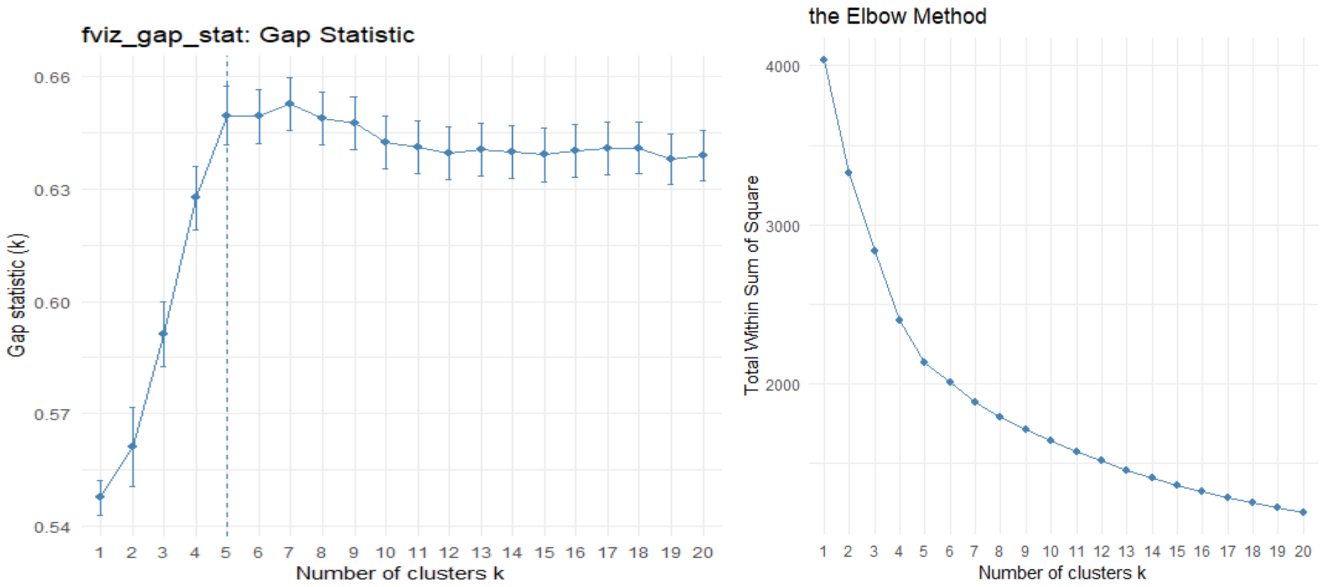

(a) **(b)**

Figure S4: Suggested number of morbidity clusters: (a) Gap-statistic method; (b) elbow method.

Table S1: Suggested number of morbidity patterns based on the Davies-Bouldin index.

Number of morbidity clusters	Davies-Bouldin index
2	1.7173
3	1.7679
4	1.6166
5	1.5041
6	1.6218
7	1.7946
8	1.7651
9	1.6904
10	1.7628
11	1.833
12	1.7679
13	1.7113
14	1.7154
15	1.636
16	1.5877
17	1.581
18	1.5666
19	1.6075
20	1.6525

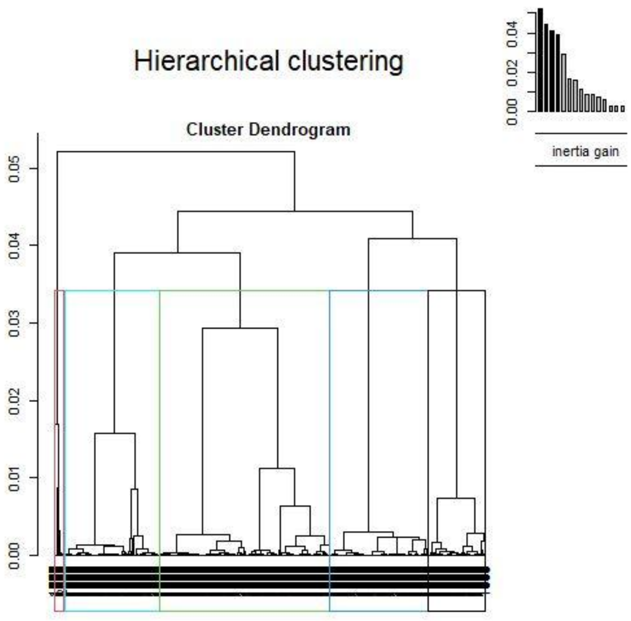

Figure S5: Dendrogram of five morbidity clusters solution

Results of Cox models.

Table S2: Cox models using attained age as timescale.

Characteristics	Number of breast cancer cases/Person-years	HR (95%CI)
**Number of morbidities**		
No morbidity	2,131/698,776	1.00 (Reference)
One morbidity	1,736/543,974	0.99 (0.92-1.05)
Two morbidities	911/266,831	1.01 (0.93-1.10)
3+ morbidities	548/161,804	0.98 (0.89-1.09)
**Morbidity patterns**		
No predominant morbidity	3,534/1,110,979	1.00 (Reference)
Psychiatric morbidities	381/115,476	1.06 (0.96-1.18)
Respiratory/immunological morbidities	611/195,129	0.99 (0.91-1.08)
Cardiovascular/metabolic morbidities	246/75,843	0.90 (0.79-1.03)
Unspecific morbidities	554/173,957	0.97 (0.89-1.07)

HR: Hazard Ratio; CI: Confidence interval

e model was adjusted for age at menarche (continuous), age at menopause (Still had periods; Had nopause before the age of 45 years; Had menopause between the age of 45 and 54; Had menopause r the age of 55), menopausal hormone therapy use (Never; Ever, less than 5 years duration; Ever, ears and longer; Ever, unknown duration), oral contraceptive use (Never; Ever, less than 10 years ration; Ever, at least 10 years duration; Ever, unknown duration; unknown status), parity and age irst birth (No live birth; At least one birth before age 30; At least one birth after age 30), BMI ntinuous), ethnicity(Asia; Black /Caribbean; White; others/unknown), Townsend score ntinuous); level of physical activity (low; moderate; high) , alcohol consumption (Never; Once or nks per week or less; Three times a week or more; Unknown status).

Table S3: Cox models using invasive breast cancer only as outcome.

Characteristics	Breast cancer cases/ Person years	HR (95%CI)
**Risk of invasive breast cancer according to the number of morbidities**		
No morbidity	1,763/698,776	1.00 (Reference)
One morbidity	1,466/543,974	1.01 (0.94-1.09)
Two morbidities	765/2,668,301	1.03 (0.95-1.13)
3+ morbidities	458/161,804	0.99 (0.89-1.11)
**Risk of invasive breast cancer according to morbidity patterns**		
No-morbidity predominant	2,943/1,110,979	1.00 (Reference)
Psychiatric morbidities	310/115,476	1.02 (0.90-1.14)
Respiratory/immunological morbidities	526/195,129	1.01 (0.92-1.11)
Cardiovascular/metabolic morbidities	209/75,843	0.93 (0.81-1.08)
Unspecific morbidities	464/173,957	0.97 (0.88-1.08)

HR: Hazard Ratio; CI: Confidence interval

The model was adjusted for age at baseline (continuous), age at menarche (continuous), age at menopause (Still had periods; Had menopause before the age of 45 years; Had menopause between the age of 45 and 54; Had menopause after the age of 55), menopausal hormone therapy use (Never; Ever, less than 5 years duration; Ever, 5 years and longer; Ever, unknown duration), oral contraceptive use (Never; Ever, less than 10 years duration; Ever, at least 10 years duration; Ever, unknown duration; unknown status), parity and age at first birth (No live birth; At least one birth before age 30; At least one birth after age 30), BMI (continuous), ethnicity(Asia; Black /Caribbean; White; others/unknown), Townsend score (continuous); level of physical activity (low; moderate; high) , alcohol consumption (Never; Once or drinks per week or less; Three times a week or more; Unknown status).

Table S4: Others modified effects.

	Morbidity patterns			Number of morbidities		
**Strata**	**Morbidity patterns**	**Cases/Person-years**	**HR (95%CI)**	**Number of morbidities**	**Cases/Person-years**	**HR(95%CI)**
**BMI**						
BMI<25						
	No predominant morbidity	1,394/474,328	1.00 (Reference)	No morbidity	1,000/343,481	1.00 (Reference)
	Psychiatric morbidities	134/40,019	1.16 (0.97-1.39)	One morbidity	605/207,588	0.97 (0.88-1.07)
	Respiratory/immunological morbidities	208/72,425	0.99 (0.85-1.14)	Two morbidities	236/77,385	0.99 (0.86-1.15)
	Cardiovascular/metabolic morbidities	25/10,757	0.74 (0.50-1.1)	3+ morbidities	107/33,666	1.02 (0.84-1.25)
	Unspecific morbidities	187/64,591	0.97 (0.83-1.13)			
25≤BMI<30						
	No predominant morbidity	1,364/413,854	1.00 (Reference)	No morbidity	787/250,485	1.00 (Reference)
	Psychiatric morbidities	136/42,618	0.99 (0.83-1.18)	One morbidity	710/209,115	1.05 (0.95-1.16)
	Respiratory/immunological morbidities	246/71,992	1.04 (0.91-1.19)	Two morbidities	343/102,742	1.01 (0.89-1.14)
	Cardiovascular/metabolic morbidities	82/25,272	0.94 (0.75-1.18)	3+ morbidities	184/56,282	0.98 (0.83-1.15)
	Unspecific morbidities	196/64,889	0.90 (0.78-1.05)			
BMI≥30						
	No predominant morbidity	776/222,798	1.00 (Reference)	No morbidity	344/104,809	1.00 (Reference)
	Psychiatric morbidities	111/32,839	1.00 (0.82-1.21)	One morbidity	421/127,272	0.97 (0.84-1.12)
	Respiratory/immunological morbidities	157/50,712	0.90 (0.76-1.07)	Two morbidities	332/86,704	1.11 (0.95-1.29)
	Cardiovascular/metabolic morbidities	139/39,814	0.98 (0.82-1.17)	3+ morbidities	257/71,856	1.03 (0.87-1.21)
	Unspecific morbidities	171/44,478	1.09 (0.92-1.29)			
**Socioeconomic status**						
Q1						
	No predominant morbidity	973/287,249	1.00 (Reference)	No morbidity	595/184,871	1.00 (Reference)
	Psychiatric morbidities	87/25,482	1.01 (0.81-1.25)	One morbidity	467/137,980	1.00 (0.89-1.13)
	Respiratory/immunological morbidities	125/47,584	0.77 (0.64-0.93)	Two morbidities	192/62,958	0.87 (0.74-1.02)
	Cardiovascular/metabolic morbidities	43/13,306	0.85 (0.62-1.15)	3+ morbidities	123/30,988	1.09 (0.89-1.33)
	Unspecific morbidities	149/43,176	0.99 (0.84-1.18)			
Q2						
	No predominant morbidity	907/280,584	1.00 (Reference)	No morbidity	574/177,872	1.00 (Reference)
	Psychiatric morbidities	103/27,248	1.17 (0.95-1.43)	One morbidity	425/135,941	0.92 (0.81-1.04)
	Respiratory/immunological morbidities	142/45,394	0.96 (0.80-1.15)	Two morbidities	233/63,553	1.04 (0.89-1.21)
	Cardiovascular/metabolic morbidities	57/16,034	0.97 (0.74-1.27)	3+ morbidities	115/35,392	0.89 (0.72-1.09)
	Unspecific morbidities	138/43498	0.95 (0.80-1.14)			

Q3						
	No predominant morbidity	871/277,253	1.00 (Reference)	No morbidity	492/174,608	1.00 (Reference)
	Psychiatric morbidities	99/29,083	1.08 (0.88-1.33)	One morbidity	463/136,119	1.15 (1.01-1.30)
	Respiratory/immunological morbidities	182/48,301	1.19 (1.01-1.39)	Two morbidities	255/66,090	1.25 (1.07-1.46)
	Cardiovascular/metabolic morbidities	50/18,628	0.77 (0.57-1.02)	3+ morbidities	145/39,530	1.15 (0.95-1.39)
	Unspecific morbidities	153/43,083	1.1 (0.93-1.31)			
Q4						
	No predominant morbidity	783/265,893	1.00 (Reference)	No morbidity	470/161,424	1.00 (Reference)
	Psychiatric morbidities	92/33,662	0.93 (0.75-1.16)	One morbidity	381/133,935	0.93 (0.81-1.07)
	Respiratory/immunological morbidities	162/53,851	1.02 (0.86-1.20)	Two morbidities	231/74,230	0.98 (0.83-1.15)
	Cardiovascular/metabolic morbidities	96/27,875	1.06 (0.85-1.31)	3+ morbidities	165/55,893	0.90 (0.75-1.07)
	Unspecific morbidities	114/44,201	0.85 (0.70-1.03)			
**Physical activity**						
Low						
	No predominant morbidity	1,144/335,252	1.00 (Reference)	No morbidity	633/196,301	1.00 (Reference)
	Psychiatric morbidities	149/41,545	1.06 (0.89-1.26)	One morbidity	592/174,028	1.01 (0.90-1.13)
	Respiratory/immunological morbidities	202/64,846	0.91 (0.78-1.05)	Two morbidities	351/97,545	1.03 (0.90-1.18)
	Cardiovascular/metabolic morbidities	106/33,471	0.86 (0.70-1.05)	3+ morbidities	226/70,797	0.90 (0.77-1.05)
	Unspecific morbidities	201/63,557	0.91 (0.78-1.06)			
Moderate						
	No predominant morbidity	1,313/403,193	1.00 (Reference)	No morbidity	813/257,892	1.00 (Reference)
	Psychiatric morbidities	137/39,848	1.06 (0.89-1.27)	One morbidity	634/196,019	0.98 (0.88-1.09)
	Respiratory/immunological morbidities	218/69,025	0.97 (0.84-1.12)	Two morbidities	319/91,282	1.03 (0.90-1.17)
	Cardiovascular/metabolic morbidities	72/23,002	0.87 (0.69-1.11)	3+ morbidities	184/49,730	1.06 (0.90-1.25)
	Unspecific morbidities	210/59,854	1.06 (0.91-1.22)			
High						
	No predominant morbidity	1,077/372,534	1.00 (Reference)	No morbidity	685/244,582	1.00 (Reference)
	Psychiatric morbidities	95/34,084	0.99 (0.80-1.22)	One morbidity	510/173,928	1.00 (0.90-1.13)
	Respiratory/immunological morbidities	191/61,258	1.08 (0.93-1.26)	Two morbidities	241/78,004	1.02 (0.88-1.19)
	Cardiovascular/metabolic morbidities	68/19,370	1.10 (0.86-1.41)	3+ morbidities	138/41,277	1.08 (0.90-1.30)
	Unspecific morbidities	143/50,547	0.96 (0.80-1.14)			
**Menopause status at baseline**						
Still had periods						
	No predominant morbidity	864/312,408	1.00 (Reference)	No morbidity	680/244,210	1.00 (Reference)
	Psychiatric morbidities	117/34,901	1.19 (0.98-1.44)	One morbidity	375/135,031	0.98 (0.86-1.11)

Respiratory/immunological morbidities	144/57,604	0.90 (0.75-1.07)	Two morbidities	125/48,051	0.90 (0.75-1.09)
Cardiovascular/metabolic morbidities	14/6,600	0.71 (0.42-1.21)	3+ morbidities	66/20,749	1.09 (0.85-1.41)
Unspecific morbidities	107/36,528	1.05 (0.86-1.29)			
Had menopause, <45					
No predominant morbidity	281/103,530	1.00 (Reference)	No morbidity	136/56,107	1.00 (Reference)
Psychiatric morbidities	37/14,554	0.94 (0.67-1.33)	One morbidity	175/57,543	1.21 (0.96-1.51)
Respiratory/immunological morbidities	70/23,487	1.09 (0.84-1.42)	Two morbidities	99/36,131	1.06 (0.81-1.37)
Cardiovascular/metabolic morbidities	39/13,976	0.95 (0.68-1.32)	3+ morbidities	88/29,705	1.11 (0.85-1.46)
Unspecific morbidities	71/23,938	1.06 (0.82-1.38)			
Had menopause, 45-54					
No predominant morbidity	2,010/597731.7	1.00 (Reference)	No morbidity	1,115/346,601	1.00 (Reference)
Psychiatric morbidities	186/57692.2	0.96 (0.82-1.11)	One morbidity	987/301,661	0.98 (0.901.07)
Respiratory/immunological morbidities	329/98088.7	0.98 (0.88-1.11)	Two morbidities	570/154,903	1.07 (0.97-1.19)
Cardiovascular/metabolic morbidities	151/46677.3	0.89 (0.75-1.06)	3+ morbidities	316/95,082	0.95 (0.83-1.08)
Unspecific morbidities	312/98056.7	0.92 (0.82-1.04)			
Had menopause, >55					
No predominant morbidity	379/97,310	1.00 (Reference)	No morbidity	200/51,858	1.00 (Reference)
Psychiatric morbidities	41/8,328	1.25 (0.90-1.72)	One morbidity	199/49,739	1.01 (0.83-1.23)
Respiratory/immunological morbidities	68/15,950	1.08 (0.83-1.39)	Two morbidities	117/27,746	1.04 (0.83-1.31)
Cardiovascular/metabolic morbidities	42/8,589	1.19 (0.86-1.64)	3+ morbidities	78/16,268	1.16 (0.89-1.51)
Unspecific morbidities	64/15,434	1.04 (0.80-1.36)			

HR: Hazard ratio; CI: Confidence interval;

Table S5: Association between breast cancer risk and the preexisting Charlson single pre-existing diseases at baseline.

Pre-existing Charlson single disease at baseline	Number of breast cancer cases/Person years	Age-adjusted model HR (95%CI)	Multivariable model HR (95%CI)
**Myocardial infarction**			
No	5,290/1,657,227	1.00 (Reference)	1.00 (Reference)
Yes	36/14,157	0.72 (0.52-1.01)	0.73 (0.53-1.02)
**Periferal vascular disease**			
No	5,307/1,662,769	1.00 (Reference)	1.00 (Reference)
Yes	19/8,615	0.66 (0.42-1.04)	0.68 (0.43-1.06)
**Cerebrovascular disease**			
No	5,249/1,645,452	1.00 (Reference)	1.00 (Reference)
Yes	77/25,932	0.87 (0.69-1.09)	0.88 (0.7-1.11)
COPD			
No	4,584/1,434,041	1.00 (Reference)	1.00 (Reference)
Yes	742/237,344	0.98 (0.91-1.06)	0.97 (0.90-1.05)
**Rheumatoid arthritis**			
No	5,227/1,635,3845	1.00 (Reference)	1.00 (Reference)
Yes	99/36,001	0.82 (0.67-1.0)	0.82 (0.67-1.0)
**Peptidic ulcer**			
No	5,235/1,646,897	1.00 (Reference)	1.00 (Reference)
Yes	91/24,488	1.11 (0.90-1.36)	1.12 (0.91-1.38)
**Mild liver disease**			
No	5,282/1,659,368	1.00 (Reference)	1.00 (Reference)
Yes	44/12,017	1.12 (0.83-1.51)	1.13 (0.84-1.51)
**Irritable bowel syndrome**			
No	5,142/1,619,052	1.00 (Reference)	1.00 (Reference)
Yes	184/52,332	1.06 (0.91-1.22)	1.03 (0.89-1.20)

HR: Hazard Ratio; CI: Confidence interval

The model was adjusted for age at baseline (continuous), age at menarche (continuous), age at menopause (Still had periods; Had menopause before the age of 45 years; Had menopause between the age of 45 and 54; Had menopause after the age of 55), menopausal hormone therapy use (Never; Ever, less than 5 years duration; Ever, 5 years and longer; Ever, unknown duration), oral contraceptive use (Never; Ever, less than 10 years duration; Ever, at least 10 years duration; Ever, unknown duration; unknown status), parity and age at first birth (No live birth; At least one birth before age 30; At least one birth after age 30), BMI (continuous), ethnicity(Asia; Black /Caribbean; White; others/unknown), Townsend score (continuous); level of physical activity (low; moderate; high) , alcohol consumption (Never; Once or drinks per week or less; Three times a week or more; Unknown status).

Table S6: Association between Charlson comorbidity index at baseline and breast cancer risk

CCI at baseline	Number of breast cancer cases/Person years	Age-adjusted model HR (95%CI)	Multivariable model HR (95%CI)
**0**	4,160/1,300,545	1.00 (Reference)	1.00 (Reference)
**1**	991/314,799	0.97 (0.90-1.04)	0.96 (0.89-1.03)
**2**	140/41,576	0.99 (0.84-1.18)	0.99 (0.83-1.17)
**≥3**	35/14,464	0.71 (0.51-10)	0.72 (0.51-10)

HR: Hazard Ratio; CI: Confidence interval

The model was adjusted for age at baseline (continuous), age at menarche (continuous), age at menopause (Still had periods; Had menopause before the age of 45 years; Had menopause between the age of 45 and 54; Had menopause after the age of 55), menopausal hormone therapy use (Never; Ever, less than 5 years duration; Ever, 5 years and longer; Ever, unknown duration), oral contraceptive use (Never; Ever, less than 10 years duration; Ever, at least 10 years duration; Ever, unknown duration; unknown status), parity and age at first birth (No live birth; At least one birth before age 30; At least one birth after age 30), BMI (continuous), ethnicity(Asia; Black /Caribbean; White; others/unknown), Townsend score (continuous); level of physical activity (low; moderate; high) , alcohol consumption (Never; Once or drinks per week or less; Three times a week or more; Unknown status).Black /Caribbean; White; others/unknown), Townsend score (continuous); level of physical activity (low; moderate; high) , alcohol consumption (Never; Once or drinks per week or less; Three times a week or more; Unknown status).
